# Systematic analysis of the lysine succinylome in the model medicinal mushroom *Ganoderma lucidum*

**DOI:** 10.1186/s12864-019-5962-0

**Published:** 2019-07-16

**Authors:** Guangyuan Wang, Lili Xu, Hao Yu, Jie Gao, Lizhong Guo

**Affiliations:** Shandong Province Key Laboratory of Applied Mycology, College of Life Sciences, Qingdao Agricultural University, Changcheng Road, No.700, Qingdao, 266109 China

**Keywords:** *Ganoderma lucidum*, Succinylome, Triterpenoids, Polysaccharides, Immunomodulatory protein, Post-translational modification

## Abstract

**Background:**

*Ganoderma lucidum*, one of the best-known medicinal mushrooms in the world, produces more than 400 different bioactive compounds. However, the regulation of these bioactive compounds biosynthesis is still unclear. Lysine succinylation is a critical post-translational modification and has many important functions in all aspects of eukaryotic and prokaryotic cells. Although it has been studied for a long time, its function is still unclear in *G. lucidum*. In this study, a global investigation was carried out on the succinylome in *G. lucidum*.

**Results:**

In total, 382 modified proteins which contain 742 lysine succinylated sites were obtained. The proteomics data are available through ProteomeXchange with the dataset accession number PXD013954. Bioinformatics analysis revealed that the succinylated proteins were distributed in various cellular biological processes and participated in a great variety of metabolic pathways including carbon metabolism and biosynthesis of secondary metabolites. Notably, a total of 47 enzymes associated with biosynthesis of triterpenoids and polysaccharides were found to be succinylated. Furthermore, two succinylated sites K90 and K106 were found in the conserved Fve region of immunomodulatory protein LZ8. These observations show that lysine succinylation plays an indispensable role in metabolic regulation of bioactive compounds in *G. lucidum*.

**Conclusions:**

These findings indicate that lysine succinylation is related to many metabolic pathways, especially pharmacologically bioactive compounds metabolism. This study provides the first global investigation of lysine succinylation in *G. lucidum* and the succinylome dataset provided in this study serves as a resource to further explore the physiological roles of these modifications in secondary metabolism.

**Electronic supplementary material:**

The online version of this article (10.1186/s12864-019-5962-0) contains supplementary material, which is available to authorized users.

## Background

*Ganoderma lucidum*, also known as “Ling Zhi or Red Reishi”, is a well-known representative of herbal mushroom that has been used extensively as traditional Chinese medicine for thousands of years. A great deal of work has demonstrated that *G. lucidum* presents various therapeutic bioactivities, such as anti-tumor, anti-bacterial, anti-atherosclerotic, antiviral (including anti-HIV), anti-inflammatory, radio protective, sleep promoting and immunomodulatory activities [1, 2]. Modern pharmacological research has identified more than 400 different bioactive compounds form the mycelia, fruiting body and spores of *G. lucidum* [3, 4]. The two major categories of bioactive compounds are triterpenoids and polysaccharides [3]. In the past few years, genome-scale metabolic models and transcriptome data have been used to elucidate the bioactive compounds mechanisms [3, 5]. These findings have provided important resources for elucidating the secondary metabolic pathways and their regulation. Nevertheless, the regulation of the biosynthesis of these bioactive compounds is still unclear.

Previous investigations have demonstrated that protein post-translational modification (PTM) has critical functions in modulating multiple intracellular metabolisms [6, 7]. PTMs can regulate the function of proteins via introducing various functional groups, e.g., phospho, acetyl, ubiquityl, methyl, malonyl, and succinyl groups [8]. Among them, lysine succinylation is a new protein modification discovered in recent years, which exhibits an important role in cellular physiology [7, 9]. Previous reports have proved that succinyl-CoA might be a common donor of succinyl group for lysine succinylation [9]. By reversibly adding succinyl groups to lysine residues, protein succinylation can regulate multiple biochemical processes such as protein localization, enzyme activity and protein stability. Similar to acetylated proteins, a large number of proteins in chloroplast, mitochondrion, cytosol, and cell nucleus have also been found to be succinylated [7, 10], indicating a wide variety of regulations by lysine succinylation.

In the past few years, lysine succinylation has been widely investigated at the level of proteomics thanks to the development of lysine-succinylated peptides immune precipitation technology. Succinylated proteins are found in large quantities in plants [7, 11], yeast [12], fungus [13], and bacteria [14–16]. These proteomic analyses revealed a wide range of roles for succinyl modification in various cellular functions such as catalytic activities, energy generation and photosynthesis in plants. However, compared with these species, there are few researches on the mushroom succinyl proteome.

*G. lucidum* produces a diverse set of useful bioactive compounds and is considered a cell factory for screening pharmacologically bioactive compounds. There are a lot of acyltransferase and deacylase homologous sequences in *G. lucidum* genome, indicating that lysine succinylation may play an important function in “Ling Zhi” development and secondary metabolites biosynthesis. To confirm this hypothesis, we performed a succinylome investigation in *G. lucidum*. We further conducted functional analyses of all the succinylated proteins and the obtained results provided a comprehensive view for the regulation of lysine succinylation in multiple biological processes, particularly in biosynthesis of bioactive metabolites.

## Results

### Proteome-scale analysis of succinylproteins in *G. lucidum*

**Fig. 1:**
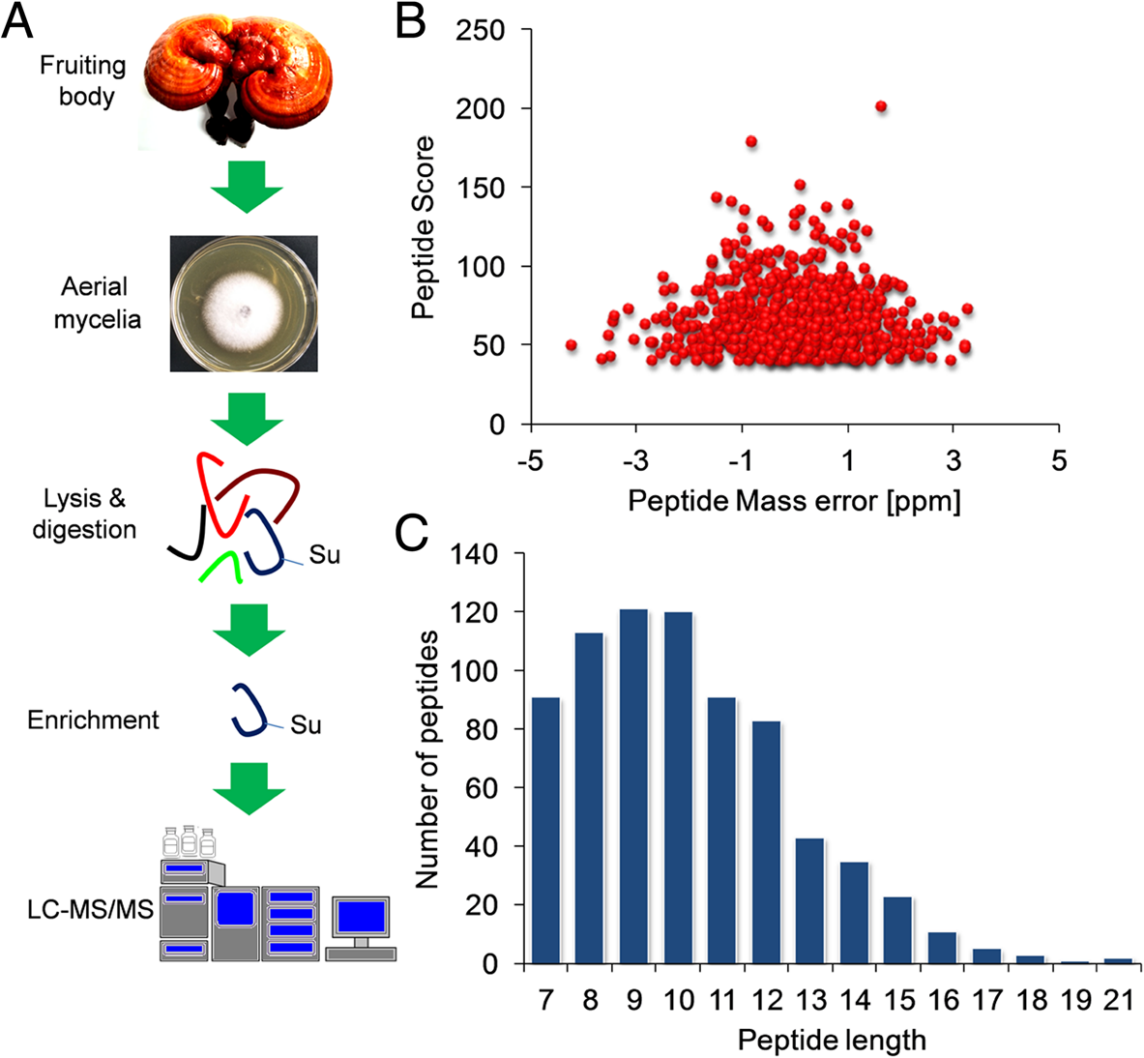
Systematic analysis of lysine succinylation sites in *G. lucidum*. **a** Work flow used in this study. **b** Distributions of mass errors for all the succinylpeptides. **c** Distributions of all the modified peptides length

To investigate the protein succinylome of *G. lucidum*, a proteomic analysis of succinylated proteins and their modification residues in *G. lucidum* was performed and the experimental process of this study was exhibited in Fig. 1a. In order to verify the mass spectrometry data, the quality errors of all the resulting peptides were detected. As shown in Fig. 1b, all the distributions of mass errors were close to zero and all the errors were less than 5 ppm, confirming that the mass spectrometry dataset obtained in this study had high accuracy. Examination of the distribution of all identified peptides was further performed and the results revealed that the number of amino acids in most of peptides was between 7 and 15 (Fig. 1c), suggesting that the prepared samples met the requirement for proteomics analysis [8, 17, 18]. The MS/MS spectra of three examples of succinylated peptides were shown in Additional file 1: Figure S1. As such, totally 742 lysine sites in 382 protein groups were found to be succinylated in *G. lucidum* (Additional file 2: Table S1). Glyceraldehyde-3-phosphate dehydrogenase (GAPDH), one of the key enzymes in the glycolysis pathway, was found to be succinylated (Additional file 2: Table S1). To confirm this succinylated protein, we performed immunoprecipitation and Western blot analysis. Consistent with the results of MS/MS, succinylation of GAPDH in *G. lucidum* was validated (Additional file 1: Figure S2).

### Conservation of succinylproteins

**Fig. 2:**
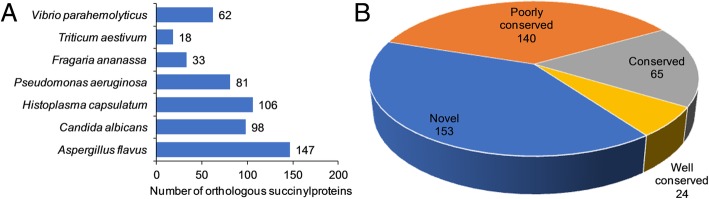
Analysis the conservation of identified succinylproteins in *G. lucidum*. **a** The number of homologous succinylproteins in 7 species. **b** A pie chart showing the conservative orthologs in *A. flavus*, *C. albicans*, *F. ananassa*, *H. capsulatum*, *P. aeruginosa*, *T. aestivum* and *V. parahemolyticus.* The group classification criteria were as follows: Well-conserved, five to seven orthologous succinylproteins; Conserved, three to four orthologous succinylproteins; Poorly conserved, one to two orthologs; Novel, zero orthologs

So far, large amounts of lysine succinylated proteins have been found in both prokaryotes and eukaryotes [7, 11–16]. However, the conservation of lysine succinylome in these species is still unclear. Therefore, in order to find the orthologs of lysine succinylated proteins in *G. lucidum*, we conducted a protein BLAST search against 7 species with determined succinylomes: *Aspergillus flavus*, *Candida albicans*, *Histoplasma capsulatum*, *Pseudomonas aeruginosa*, *Fragaria ananassa*, *Triticum aestivum* and *Vibrio parahemolyticus.* A total of 545 orthologous succinylproteins were found in these seven species (Fig. 2a and Additional file 2: Table S2). As shown in Fig. 2a and Additional file 2: Table S2, 229 succinylated proteins in *G. lucidum* had orthologs in *V. parahemolyticus* (62 proteins), *T. aestivum* (18 proteins), *F. ananassa* (33 proteins), *P. aeruginosa* (81 proteins), *H. capsulatum* (106 proteins), *C. albicans* (98 proteins) and *A. flavus* (147 proteins), which account for 60% (229/382 proteins) of the total succinylproteins in *G. lucidum*. According to the number of homologous proteins in different species, the homologous succinylated proteins in *G. lucidum* were classified. As shown in Fig. 2b, 6.3% of the total succinylproteins (24/382 proteins) belonged to the well-conserved category, which had orthologs in 5 to 7 species. The percentage of conserved proteins containing 3 to 4 orthologs was 17% of the total succinylproteins (65/382 proteins) (Fig. 2b). Interestingly, 36.6% (140/382 proteins) and 40.1% (153/382 proteins) of the succinylated proteins in *G. lucidum* were grouped as poorly conserved proteins which contained 1 to 2 homologous proteins and novel proteins without orthologs (Fig. 2b), respectively. These results indicate that although succinylproteins are conserved in different species, many organisms still contain unique succinylproteins with specific functions.

### Pattern analysis of lysine succinylated sites

**Fig. 3:**
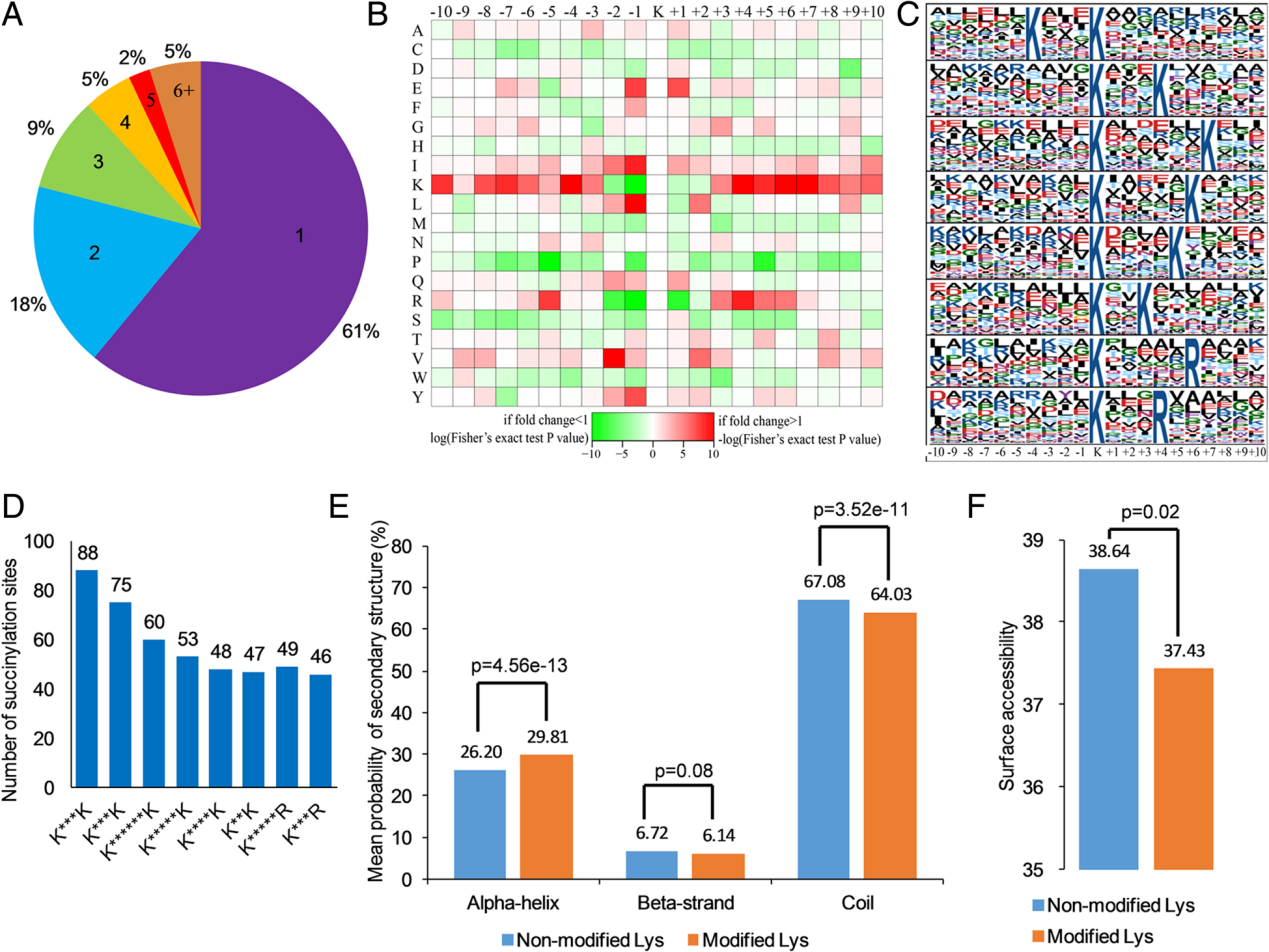
Properties of the modified peptides in *G. lucidum*. **a** A pie chart showing the percentage and number of succinylated residues per protein. **b** A heat map of the amino acid compositional frequencies surrounding the succinylated residues. **c** The modified peptides motifs containing ±10 amino acids surrounding the identified residues. **d** Number of succinylation sequence motifs. **e** Analysis the secondary structure of succinylproteins. **f** Predicted surface accessibility of succinylated residues

Proteins can be modified by succinylation on one or more lysine sites. Therefore, we counted the number of succinylated residues in each modified protein in *G. lucidum*. As shown in Fig. 3a, 61% (233) of the identified proteins had only one lysine succinylated residue and the proportions of proteins containing two, three, four, five and six or more lysine succinylated residues were 18% (69/382 proteins), 9% (35/382 proteins), 5% (18/382 proteins), 2% (8/382 proteins) and 5% (19/382 proteins), respectively.

It has been well documented that the modification positions have preference for lysine at specific sites [8, 17–20]. The amino acid compositional frequencies around the modified lysine were therefore investigated. As shown in Fig. 3b, lysine (K), arginine (R), leucine (L) and valine (V) had the highest frequency in positions − 10 to + 10, while proline (P) had the lowest frequency. The amino acid sequences of succinylated peptides were further studied by Motif-X program. Consistent with the results of amino acid heat map (Fig. 3b), eight conserved motifs, K***K_su_, K_su_***K, K_su_******K, K_su_*****K, K_su_****K, K_su_**K, K_su_*****R and K_su_***R (K_su_ indicates the modified lysine site and * indicates a random amino acid site), were obtained (Fig. 3c). These conserved sequences matched to 466 identified modified peptides, exhibiting different abundances (Fig. 3d and Additional file 2: Table S3). Therefore, proteins with K or R in corresponding sites are more easily to be the preferred substrates of lysine succinyltransferase in *G. lucidum*. It is noteworthy that the conserved motifs form strawberry stigmata [11], common wheat [7], *C. albicans* [12], and *V. parahemolyticus* [14] are different, indicating that different species contain unique succinylated proteins with specific functions.

In order to explore the relationship between the protein secondary structure and the modified lysine residues, the secondary structure of all the succinylated proteins in *G. lucidum* was analyzed. The results showed that the succinylated lysines were more frequently found in alpha-helix (*p* = 4.56e-13) and coil (*p* = 3.52e-11), and less frequently in beta-strand (*p* = 0.08) (Fig. 3e). We further evaluated the succinylated lysine sites for solvent accessibility and the results revealed that 38.64% of non-modified lysine residues were located on the protein surface (*p* = 0.02) compared with 37.43% of the modified lysine sites (Fig. 3f). These results suggest that the surface properties of modified proteins in *G. lucidum* may be altered by succinylation.

### Succinylated proteins functional annotation

**Fig. 4:**
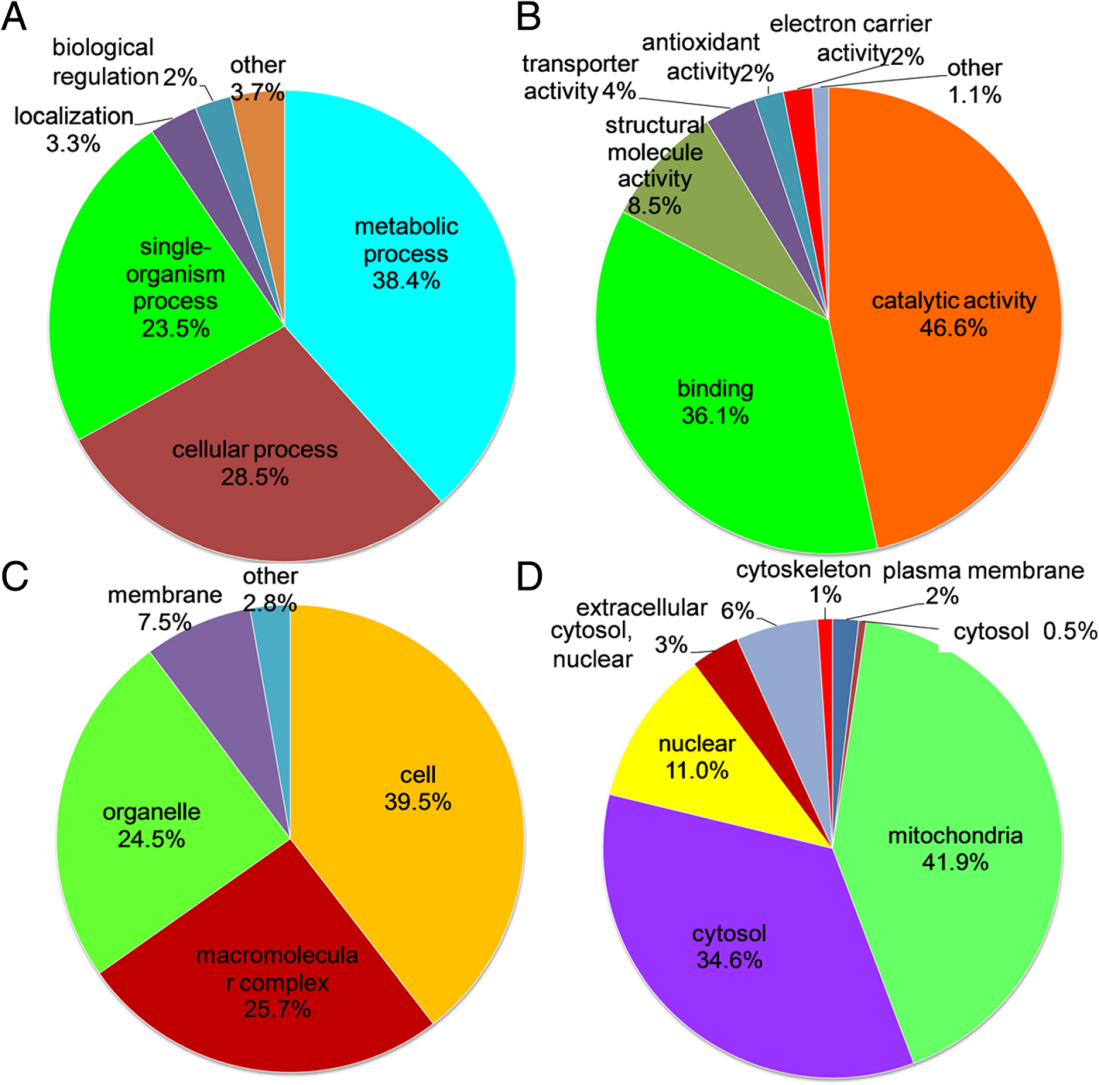
Functional classification for the identified succinylproteins in *G. lucidum*. **a** Classification of succinylproteins based on their biological processes. **b** Classification of succinylproteins according to their molecular functions. **c** Classification of succinylproteins based on their cellular components. **d** Analysis the intracellular locations of the identified succinylproteins

In order to further understand the potential roles of succinylproteins in *G. lucidum*, all the identified proteins were annotated by Gene Ontology (GO) functional classification in terms of their biological processes, cell compositions and molecular functions. Based on the biological process analysis in *G. lucidum*, it was found that 230 proteins in the metabolic process, 171 proteins in the cellular process and 141 proteins in the single-organism process were modified by succinylation (Fig. 4a and Additional file 2: Table S4), respectively. In accordance with the above findings, a high proportion of the identified succinylproteins were related to catalytic activity (46.6%), binding (36.1%) and structural molecule activity binding (8.5%) according to molecular function analysis (Fig. 4b and Additional file 2: Table S4). These observations are consistent with the previous results in *T. aestivum* [7] and *C. albicans* [12]. Cellular component analysis revealed that succinylproteins were mainly distributed in cells (39.5%), macromolecular complexes (25.7%), organelles (24.5%) and membranes (7.5%) (Fig. 4c and Additional file 2: Table S4). These findings indicate that the molecular function of proteins may be changed by lysine succinylation, which may further affect various biological processes in *G. lucidum*.

The subcellular localization of all the succinylproteins was also investigated. As shown in (Fig. 4d and Additional file 2: Table S5), a large proportion of the identified proteins located in mitochondria (41.9%) and cytosol (34.6%). It is noteworthy that 42 (11.0%) succinylated proteins, such as Histone H2B, Histone H3, and Histone H4, were found to be distributed in the nuclear (Fig. 4d and Additional file 2: Table S5), suggesting that lysine succinylation has the function of transcriptional regulation in *G. lucidum*. In addition, the results in (Fig. 4d and Additional file 2: Table S5) also demonstrated that the percentages of succinylproteins distributed in extracellular space, plasma membrane and cytoskeleton were 6% (22 proteins), 2.0% (7 proteins) and 1% (4 proteins), respectively. These observations reveal that the modified succinylproteins have extensive biological functions in *G. lucidum*.

### Functional enrichment analysis

**Fig. 5:**
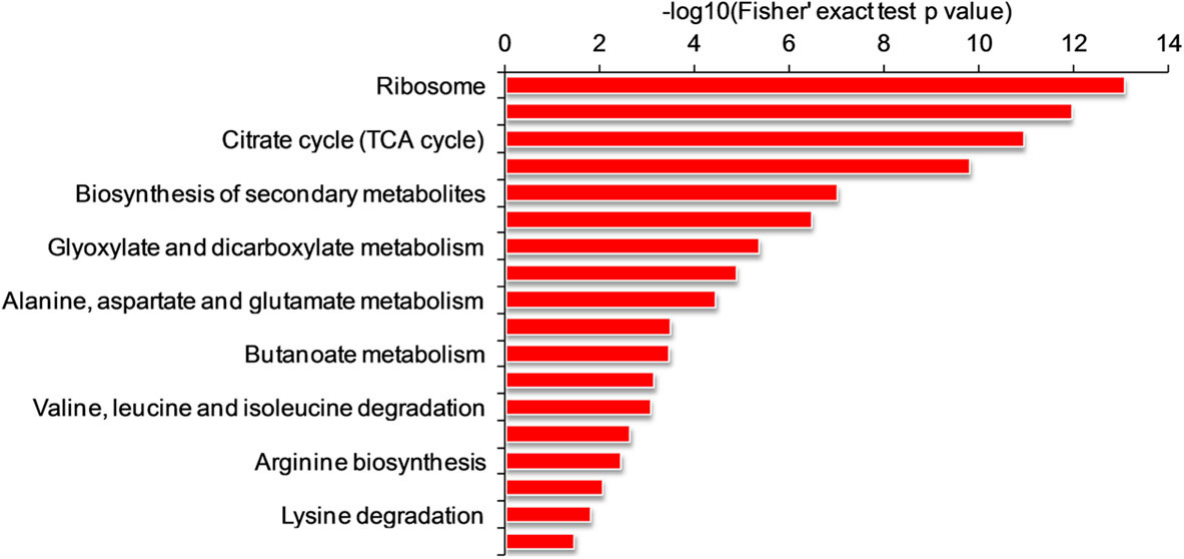
Enrichment analysis of succinylproteins based on KEGG pathway in *G. lucidum*

In order to reveal the preferred target protein types of lysine succinylation, we conducted a functional enrichment analysis of the obtained succinylome via GO, Kyoto Encyclopedia of Genes and Genomes (KEGG) pathway and protein domains, respectively. In GO biological process category, many succinylproteins were involved in biosynthetic process, translation and metabolic process (Additional file 1: Figure S3 and Additional file 2: Table S6). Consistent with these observations, it was found that the proteins associated with structural molecular activities, ribosome structure composition, and oxidoreductase activities were highly enriched through enrichment analysis of GO molecular functions (Additional file 1: Figure S3 and Additional file 2: Table S6). Consistently, according to the GO cellular component category, the proteins located in cytoplasm, ribosome and intracellular part were more likely to be succinylated (Additional file 1: Figure S3 and Additional file 2: Table S6). To support of these findings, it was found that a lot of succinylated proteins were also significantly enriched in ribosome, carbon metabolism, biosynthesis of secondary metabolites and oxidative phosphorylation according to KEGG pathway enrichment analysis (Fig. 5 and Additional file 2: Table S7). Similar findings were also observed in the analysis of protein domain enrichment. As shown in (Additional file 1: Figure S4 and Additional file 2: Table S8), the proteins with domains of translation protein SH3-like domain, NAD(P)-binding domain, thioredoxin-like fold, biotin/lipoyl attachment, succinyl-CoA synthetase-like, binding and transferase domains were significantly enriched. Collectively, the succinylated proteins were found to be significantly enriched in diverse types of proteins and involved in multiple pathways, suggesting a critical role of lysine succinylation in cell metabolism.

### Protein-protein interactions (PPI) network of succinylproteins in *G. lucidum*

**Fig. 6:**
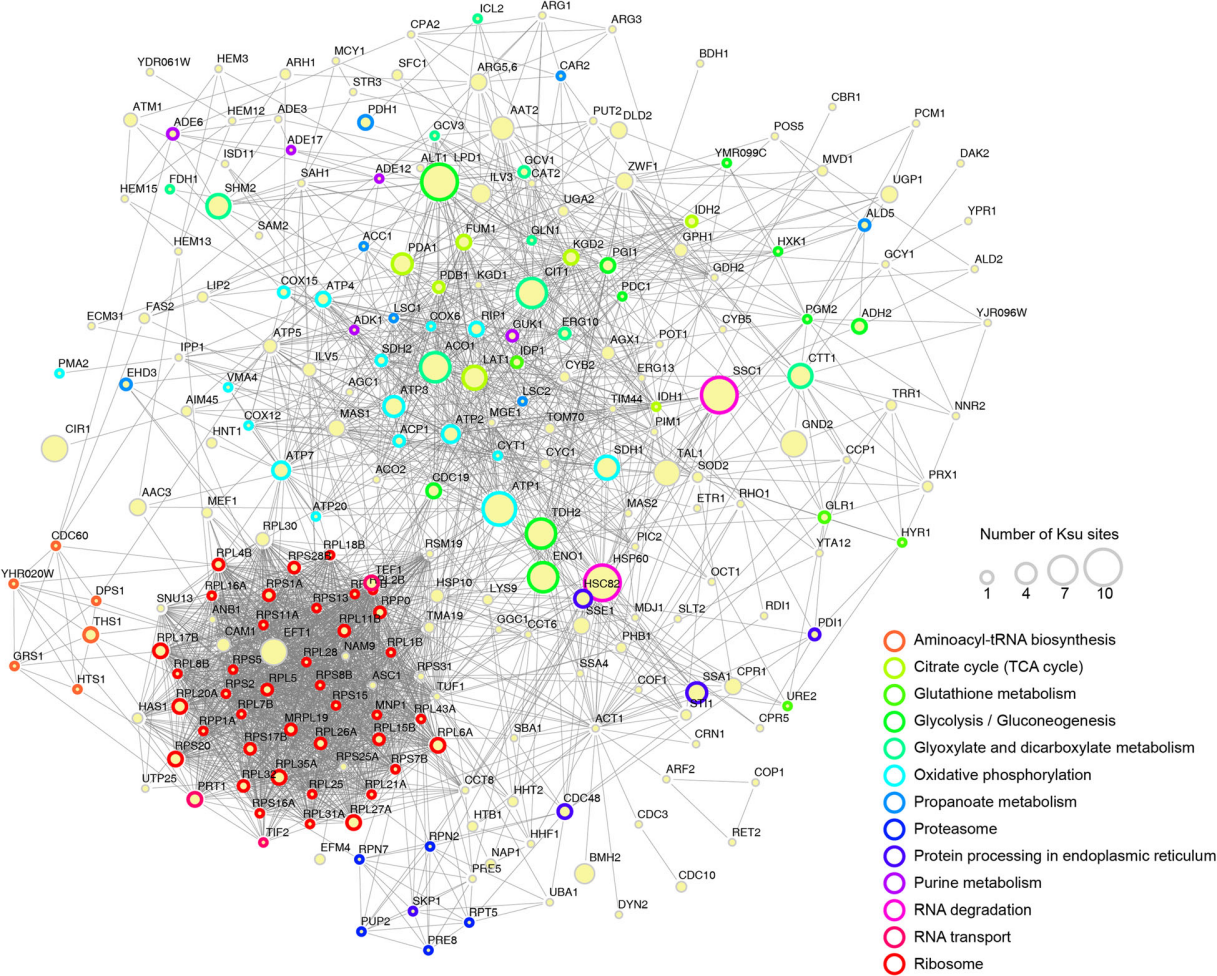
PPI network of the succinylproteins in *G. lucidum*

To reveal how the identified proteins are related to multiple interaction pathways, a PPI network of the succinylproteins was constructed [21, 22]. As shown in (Fig. 6 and Additional file 2: Table S9), a total of 275 succinylproteins were found to be mapped in PPI network database, which exhibited a global view of how the identified succinylproteins were involved in various kinds of pathways in *G. lucidum*. On the basis of the algorithm in Cytoscape software, we retrieved 13 highly interconnected clusters of succinylated proteins and these clusters extracted above included the proteins associated with aminoacyl-tRNA biosynthesis, citrate cycle, glutathione metabolism, glycolysis/gluconeogenesis, oxidative phosphorylation, purine metabolism, glyoxylate and dicarboxylate metabolism, propanoate metabolism, ribosome, RNA transports, RNA degradations, proteasome and proteins process in endoplasmic reticulum (Additional file 2: Table S9 and Fig. 6). The complicated protein interaction networks of succinylproteins suggest that the physiological interaction of these succinylproteins may contribute to the global regulation of lysine succinylation in *G. lucidum*.

### Succinylated proteins related to the biosynthesis of bioactive compounds in *G. lucidum*

**Fig. 7:**
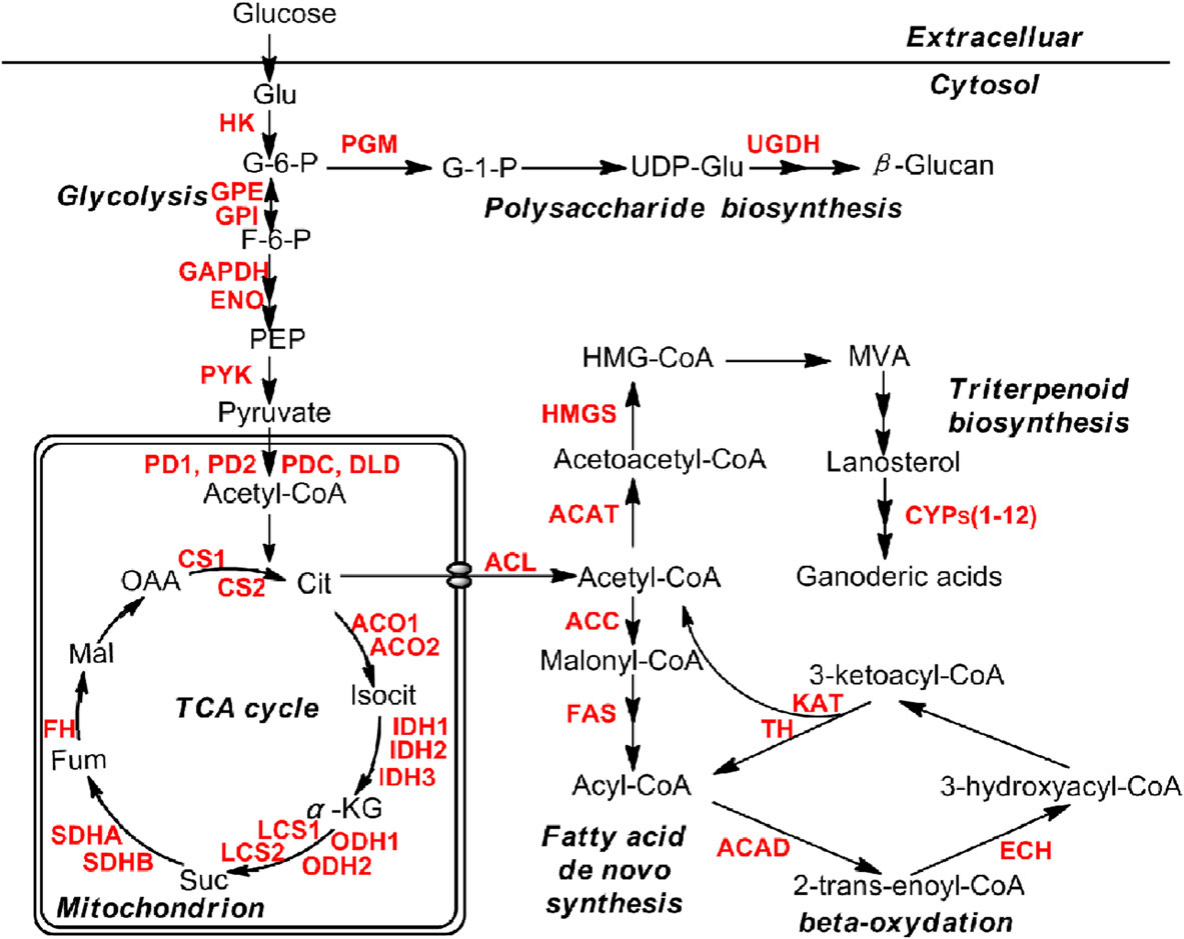
Working scheme of triterpenoid and polysaccharide biosynthesis. The succinylated proteins were highlighted in red. Enzyme annotations are included in Additional file 2: Table S10

According to the results of functional enrichment of the identified succinylproteins, the proteins related to oxidative phosphorylation, carbon metabolism, and biosynthesis of secondary metabolite were found to be much enriched (Fig. 5). These findings indicated that lysine succinylation may perform critical functions in biosynthesis of bioactive compounds in *G. lucidum*. To validate these findings, we further analyzed the succinylated proteins related to the triterpenoids and polysaccharides biosynthesis, two of the most important pharmacologically active compounds in *G. lucidum*. In agreement with the hypothesis mentioned above, a total of 47 enzymes associated with triterpenoids and polysaccharides biosynthesis were found to be succinylated (Fig. 7 and Additional file 2: Table S10). Krebs cycle, glycolysis, and fatty acid metabolism provide compounds for the biosynthesis of triterpenoids and polysaccharides. As shown in Fig. 7, a large number of enzymes in glycolysis, tricarboxylic acid cycle, fatty acid synthesis and degradation are subjected to succinylation supporting the idea that succinylation may be associated with intracellular metabolism at multiple levels.

**Fig. 8:**
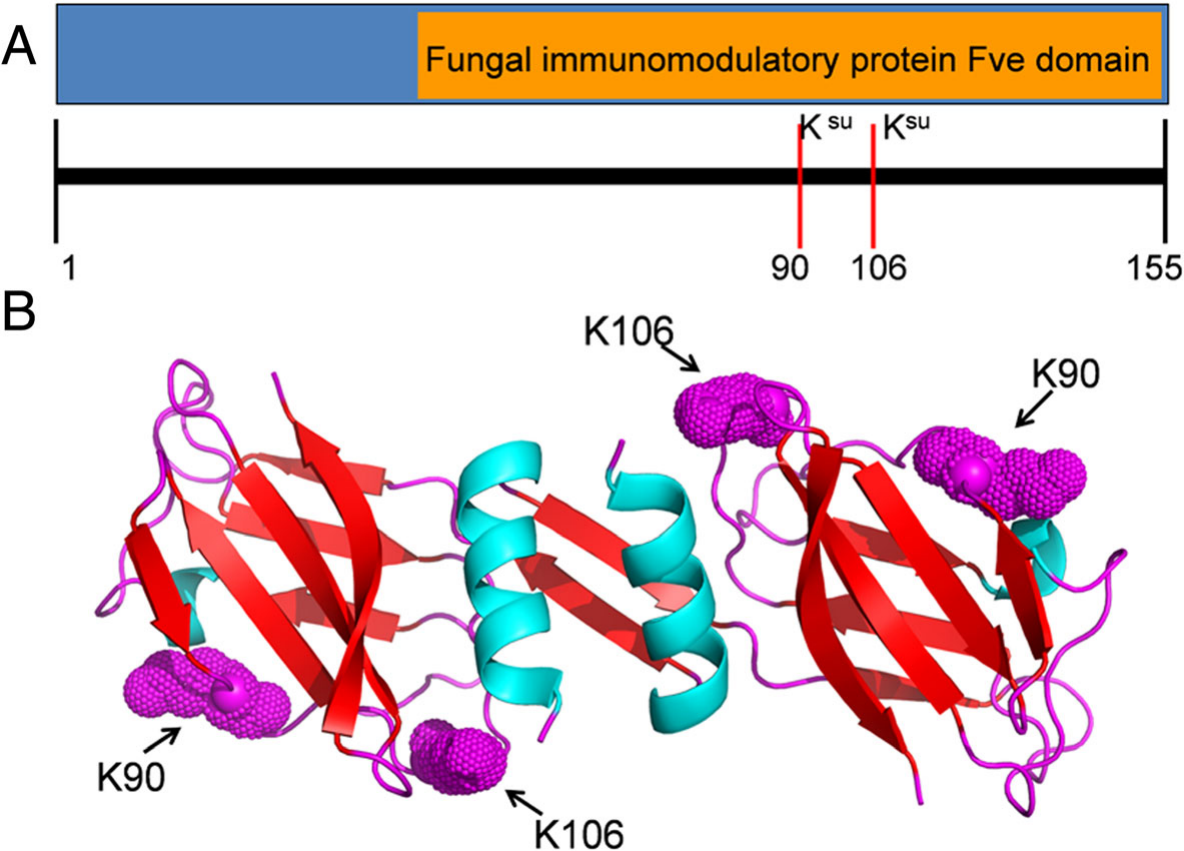
The structure of immunomodulatory protein LZ8. **a** An overview of succinylation sites in LZ8. **b** Three-dimensional structure of LZ8-LZ8 homodimer with succinylated sites K90 and K106. The structure was modeled from PDB database

Furthermore, our results also showed that two lysine residues (K90 and K106) in Fve domain of immunomodulatory protein Ling Zhi-8 (LZ8) were identified as succinylated sites (Fig. 8a and Additional file 2: Table S1). Previous studies have demonstrated that Fve is a non-covalently linked homodimer [23]. The three-dimensional structure of LZ8 with identified succinylated sites was modeled and the results showed that the succinylated lysine residues were located on the protein surface (Fig. 8b). Protein succinylation on a lysine site will change the charge states from + 1 to − 1 [9]. Therefore, Lysine succinylation is likely to lead to significant chemical properties changes of LZ8 and further changes its structure and function.

## Discussion

As one of the most representative of herbal mushroom, *G. lucidum* has been used for thousands of years in the world. To date, over 400 different bioactive compounds have been identified in *G. lucidum* [3]. However, the understanding of the regulation in these bioactive compounds biosynthesis is still very limited. Lysine succinylation is a highly conserved PTM, which widely exists in eukaryotes and prokaryotes and has many functions [7, 9]. In the present work, a proteomic study of succinylproteins in *G. lucidum* was performed. The number of succinylated proteins obtained above in *G. lucidum* was higher than that in strawberry stigmata [11] and common wheat [7], but less than that in *V. parahemolyticus* [14], *H. capsulatum* [15], *P. aeruginosa* [16] and *C. albicans* [12]. To the best of our knowledge, these data obtained in this study show the first report of succinylome in *G. lucidum*.

*G. lucidum* produces many bioactive oxygenated triterpenoids, antitumor and hypoglycemic polysaccharides and immunomodulatory protein LZ-8 [4]. The metabolic processes of these bioactive substances are related to secondary metabolism. Our succinylome functional analysis revealed a large assortment of succinylated proteins involved in secondary metabolism, showing a necessary role of lysine succinylation in these metabolisms. Other PTMs, such as acetylation and malonylation, are also involved in secondary metabolic processes in plant [8], bacteria [14, 17] and fungi [18, 20].

So far, more than 120 kinds of triterpenoids have been identified from *G. lucidum* [4], Among them, ganoderic acids (GAs) are the most valuable triterpenoids. GAs are synthesized via the mevalonic acid (MVA) pathway [3]. It is generally believed that acetyl-coenzyme A (Acetyl-CoA) is the biosynthetic feedstock of terpenoids [24]. As shown in Fig. 7, the first enzyme in this pathway is acetyl-CoA acetyltransferases (ATAC), which converts two units of acetyl-CoA to acetoacetyl CoA. Previous reports have proved that hydroxymethylglutaryl coenzyme A (HMG-CoA) is a key component in the GAs biosynthetic pathway [24]. HMG-CoA synthase (HMGS) catalyzes the conversion of acetoacetyl-CoA to HMG-CoA, which is a key step in the biosynthesis of triterpenes. Our results showed that these two key enzymes, ATAC and HMGS were both modified by succinyl groups (Fig. 7). Lanosterol is a common cyclic intermediate of triterpenoids in *G. lucidum*. However, the steps form lanosterol to GAs including a series of oxidation, reduction, isomerization, conjugation and acylation reactions are largely unknown [3, 24]. It has been well documented that the proteins in cytochrome P450 superfamily (CYPs) have significant roles in the oxidation of the lanosterol skeleton [3]. A total of 219 CYP proteins have been identified in *G. lucidum* [3]. Among them, twelve succinylated proteins were identified by the mass spectrometry (Fig. 7). CYP proteins generally have critical roles in primary and secondary metabolism [3]. Therefore, lysine succinylation may play a regulatory role at multiple levels in the biosynthesis of GAs.

Another major bioactive constituent in *G. lucidum* has been identified as polysaccharides (β-1,3-glucan and β-1,6-glucan), which exhibit various bioactivities, such as anti-oxidant, anti-tumor, anti-diabetes and immunomodulation [3, 25, 26]. Nine β-glucan biosynthesis-associated proteins have been found in *G. lucidum* [3]. Among them, two enzymes, phosphoglucomutase (PGM) and UDP-glucose 6-dehydrogenase (UGDH) were identified to be modified by succinylation (Fig. 7). Up to now, approximately 700 species of medicinal mushrooms can produce biologically active polysaccharides [27, 28]. The polysaccharides biosynthesis-associated proteins are well conserved in fungi such as *G. lucidum, Phanerochaete chrysosporium*, *Saccharomyces cerevisiae* and *Postia placenta* [3]. Lysine succinylation participated in the β-glucan biosynthesis suggesting its importance in fungal polysaccharides metabolism.

Immunomodulatory protein LZ8 was firstly isolated from *G. lucidum* in 1989 [29, 30]. Previous investigations have demonstrated that LZ8 has various bioactivities, including anti-tumor and immunomodulatory activities [31–33]. LZ8 contains an Fve domain, which is a common structure in all the fungal immunomodulatory proteins [3]. Immunomodulatory protein LZ8 was also found to be modified by succinyl groups on the lysine residues, K90 and K106 (Fig. 8). All these observations support the hypothesis that protein succinylation plays an important role in bioactive compounds biosynthesis.

## Conclusions

In summary, the provided succinylome dataset in this study illuminates a crucial role of lysine succinylation in *G. lucidum*. A great number of biological processes and biological functions were found to be involved in lysine succinylation. The identification of numerous succinylated proteins involved in pharmacologically bioactive compounds metabolism will accelerate the discovery of the complicated regulation of bioactive compounds biosynthesis in *G. lucidum* and likely in all medicinal mushrooms.

## Methods

### Strains, media and culture

*G. lucidum* G.260125–1, a monokaryotic strain, was derived from *G. lucidum* CGMCC5.0026 by protoplasting [3], and was kindly provided by Prof. Chao Sun at Chinese Academy of Medical Sciences. The strain G.260125–1 was used for lysine succinylome analysis in this study and was preserved at 4 °C in potato dextrose agar (PDA) slants composed of 200 g/l potato, 20 g/l glucose and 20 g/l agar. The strain G.260125–1 was incubated on PDA plates at 26 °C for 5 d. After that, aerial mycelia in the plates were harvested.

### Protein extraction

Protein in the strain G.260125–1 was extracted according to the procedures described [6, 34]. Briefly, the mycelia were firstly frozen in liquid nitrogen and ground. The resulting powder of mycelia was then moved into 10 ml lysis buffer which contained 0.05 M nicotinamide (NAM), 8 M urea, 0.01 M dithiothreitol (DTT), 3 μM trichostatin A (TSA), and 0.1% protease inhibitor cocktail, followed by sonicating 3 times using an ultrasonic processor [34]. The mycelia debrises were discarded by centrifuging at 16,000×g and 4 °C for 30 min. And then 15% tricarboxylic acid (TCA) was added to the supernatant at a volume ratio of 13:7. To get enough protein precipitation, the mixture was left at 4 °C for 6 h. The protein precipitation obtained above was washed for 3 times using ice-cold acetone. And then, the extracted proteins were re-dissolved with a buffer containing 8 M urea and 0.1 M NH_4_CO_3_.

### Trypsin digestion

The protein prepared as described in the previous section was chemically reduced using 10 mM dithiothreitol (DTT) for 30 min at 37 °C, followed by alkylation using 11 mM iodoacetamide (IAA) in the dark for 25 min at room temperature [34]. Urea concentration in the sample prepared above was diluted to less than 2 M with 0.1 M ammonium carbonate. Trypsin digestion was then performed as previously described [8, 17]. In brief, trypsin was firstly added into the sample solution in mass proportion of 1:50 (enzyme/protein). After digestion for 12 h, a second trypsin hydrolysis was performed. The second hydrolysis condition was 1:100 mass ratio of enzyme and protein, and the digestion time was 4 h.

### Affinity enrichment

The peptides obtained by enzymatic hydrolysis in the previous section were separated into 80 fractions by the high pH reversed phase HPLC system equipped with an Agilent 300Extend C18 column (4.6 × 250 mm, 5 μm particles) according to the methods described [7]. Afterward, the isolated samples were combined into six fractions and lyophilized. The pan succinyllysine antibody beads (PTM Biolabs) were used to enrich the succinylated peptides in the combined fractions as described [7, 11]. Trifluoroacetic acid (TFA) (0.1%) was used to release the bound peptides on the antibody beads. Finally, the obtained samples were cleaned by the C18 ZipTips system (Merck).

### Liquid chromatography-mass spectrometry (LC-MS/MS) analysis

The peptides prepared in the previous section were analyzed by the EASY-nLC 1000 UPLC, which was equipped with a reversephase analytical Acclaim PepMap RSLC column (75 μm × 150 mm, 3 μm particles, ThermoFisher) [7, 11]. The peptides MS/MS analysis was carried out by tandem MS/MS in Quadrupole-Orbitrap mass spectrometer (Q Exactive™, ThermoFisher) coupled online to UPLC system. The resolution of intact peptides in the Orbitrap was 70,000 and the resolution of ion fragments in the Orbitrap was 17,500. The normalized collision energy (NCE) was set at 28. In the MS survey scanning, a data-dependent program was executed that alternated between 1 scan and subsequent 20 scans for the top 20 precursor ions which exceeded ion number of 5E3 with dynamic exclusion of 15.0 s [8, 35]. The overfilling of the Quadrupole-Orbitrap was realized by automatic gain control (AGC). For MS scanning, the scanning range of mass spectrum was 350 ~ 1800. The fixed first mass was set at 100 m/z [17, 18, 20, 35]. The electrospray voltage of MS/MS was set at 2.0 kV.

### Data analysis

The obtained MS/MS spectra were processed by MaxQuant as described [7, 8, 36]. In brief, the resulting MS/MS data were searched against the database of *G. lucidum* coupled with a reversed decoy database [3]. The cleavage enzyme was Trypsin/P, and at most, 4 missing cleavages were allowed [8]. The errors of mass spectrum for precursor ion and fragment ion were set at 10 ppm and 0.02 Da, respectively. Cysteine carbamidomethylation was designated as the fixed modification. Lysine succinylation and methionine oxidation were designated as the variable modifications. The thresholds of false discovery rates (FDRs) for proteins, peptides and modified sites were all designated as 0.01, and seven amino acid residues was designated as the minimum length of peptide [11]. The localization probabilities of succinylated sites were set as ≥75% [8].

### Bioinformatics analysis

GO annotation of the resulting succinylome was obtained from UniProt-GOA database (http://www.ebi.ac.uk/GOA/) as previously described [7, 37]. Domain functional description of the succinylated proteins was annotated from InterPro (http://www.ebi.ac.uk/interpro/) and the metabolic pathways associated with the succinylated proteins were derived form KEGG database [7, 38]. The *p*-value of GO, protein domain and KEGG pathway in each cluster was less than 0.05, which was considered to be significant [7]. Wolfpsort (PSORT/PSORT II) was employed to analyze the intracellular locations of the proteins obtained by mass spectrometry [39]. Software Motif-x was employed to derive the sequence models around each modified site in all protein sequences, including ten residues upstream and downstream of the modified site and NetSurfP was employed to characterize the secondary structure of all the modified proteins [8, 40]. The PPI of the modified proteins was characterized using Cytoscape and a PPI network involved in the succinylated proteins was obtained from STRING database [21, 22]. The conservation of the modified proteins between *G. lucidum* and other organisms was determined using BLASTP [8].

### Western blot analysis

The mycelia of *G. lucidum* G.260125–1 were disrupted and the soluble proteins were prepared [6]. The extracted proteins (1 mg) were incubated with or without 1 μg of GAPDH antibody (Sigma-Aldrich) at 4 °C for 6 h. The mixture was then supplemented with 20 μl protein A agarose beads (GE Healthcare) followed by incubating at 4 °C for 12 h. After separated from the mixture by a centrifugation at 4 °C and 6,000×g for 1 min, the agarose beads were washed for 3 times, and the boiled SDS-PAGE sample buffer was used to elute the binding proteins on the agarose beads [7]. The eluted proteins were isolated on 12% gel using SDS-PAGE and then electrotransferred onto a polyvinylidene difluoride membrane followed by detection using GAPDH antibody (1:10,000 dilution) and succinyllysine antibody (1:2000 dilution, PTM Biolabs) [6, 7], respectively. Finally, the Western blot signal was detected using an immunoblotting detection kit (ThermoFisher).

## Additional files

Additional file 1: Three mass spectrometry examples of the succinylpeptides (**Figure S1**); Western blotting analysis with GAPDH antibody and succinyllysine antibody (**Figure S2**); GO-based enrichment analysis (**Figure S3**); Domain enrichment analysis of the succinylproteins (**Figure S4**). (DOC 9793 kb)

Additional file 2: The identifed succinylated peptides in *G. lucidum* (**Table S1**); Conservative analysis of the succinylated proteins (**Table S2**); Analysis the sequence motifs of the succinylpeptides (**Table S3**); GO functional annotation of the succinylproteins (**Table S4**); Subcellular localizations of the succinylated proteins (**Table S5**); The related proteins based on GO enrichment analysis (**Table S6**); The modified proteins based on KEGG pathway enrichment analysis (**Table S7**); The succinylated proteins based on domain enrichment analysis (**Table S8**); The proteins obtained from PPI network analysis (**Table S9**); Lysine succinylated enzymes related to triterpenoid and polysaccharide metabolism (**Table S10**). (XLSX 280 kb)

## Data Availability

The datasets supporting the results of this article are included within the article and additional files. The mass spectrometry proteomics data have been deposited to the ProteomeXchange Consortium via the PRIDE partner repository with the dataset identifier PXD013954.

## Abbreviations

Acetyl-CoA: Acetyl-coenzyme A
AGC: Automatic gain control
ATAC: Acetyl-CoA acetyltransferases
CYPs: Cytochrome P450 superfamily
DTT: Sithiothreitol
EDTA: Ethylenediaminetetraacetic acid
FDR: False discovery rate
GAPDH: Glyceraldehyde-3-phosphate dehydrogenase
GAs: Ganoderic acids
GO: Gene Ontology
HMG-CoA: Hydroxymethylglutaryl-CoA
HMGS: HMG-CoA synthase
IAA: Iodoacetamide
KEGG: Kyoto Encyclopedia of Genes and Genomes
LC-MS/MS: Liquid chromatography-mass spectrometry
LZ8: Ling Zhi-8
NAM: Nicotinamide
NCE: Normalized collision energy
NSI: Nanospray ionization
PDA: Potato dextrose agar
PGM: Phosphoglucomutase
PPI: Protein-protein interactions
PTMs: Post-translational modifications
TCA: Tricarboxylic acid
TFA: Trifluoroacetic acid
TSA: Trichostatin A
UGDH: UDP-glucose 6-dehydrogenase
