# Supplementary material for: Systematic analysis of the lysine succinylome in the model medicinal mushroom Ganoderma lucidum
Source: BMC Genomics. 2019 Jul 16;20:585. doi: 10.1186/s12864-019-5962-0 (PMC6636155; doi:10.1186/s12864-019-5962-0)
Supplement: Supplementary file 1 — Three mass spectrometry examples of the succinylpeptides (Figure S1); Western blotting analysis with GAPDH antibody and succinyllysine antibody (Figure S2); GO-based enrichment analysis (Figure S3); Domain enrichment analysis of the succinylproteins (Figure S4). (DOC 9793 kb) [file 12864_2019_5962_MOESM1_ESM.doc]

**Systematic analysis of the lysine succinylome in the model medicinal mushroom**

***Ganoderma lucidum***

Guangyuan Wang, Lili Xu, Hao Yu, Jie Gao, Lizhong Guo^*^

Shandong Province Key Laboratory of Applied Mycology, College of Life Sciences, Qingdao Agricultural University, Qingdao 266109, China

**Figure S1.** Three mass spectrometry examples of the succinylpeptides. The peptides were from GL20259-R1_1, GL22966-R1_1 and GL24555-R1_1, respectively. The succinylated (su) lysine residues, 356, 136 and 46 were indicated.

**Figure S2.** Western blotting analysis with GAPDH antibody and succinyllysine antibody. Immunoprecipitation of GAPDH was performed with (+) or without (-) GAPDH antibody (Ab) and the eluted proteins were probed with either anti-succinyl lysine antibody (suK) or GAPDH antibody

**Figure S3.** GO-based enrichment analysis in terms of cell component (blue bars), molecular function (green bars), and biological process (red bars)

**Figure S4.** Domain enrichment analysis of the succinylproteins.

**Figure S1.** Three mass spectrometry examples of the succinylpeptides. The peptides were from GL20259-R1_1, GL22966-R1_1 and GL24555-R1_1, respectively. The succinylated (su) lysine residues, 356, 136 and 46 were indicated.

**
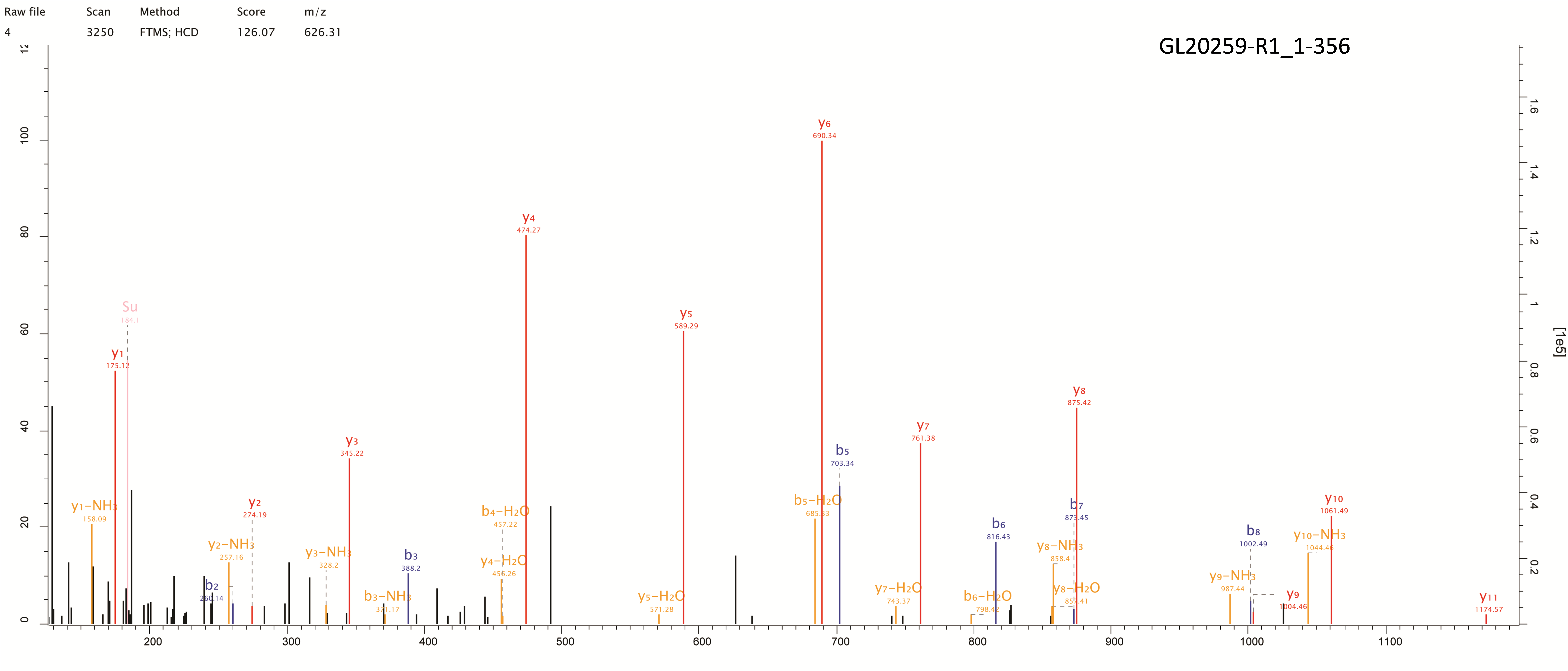
**

**
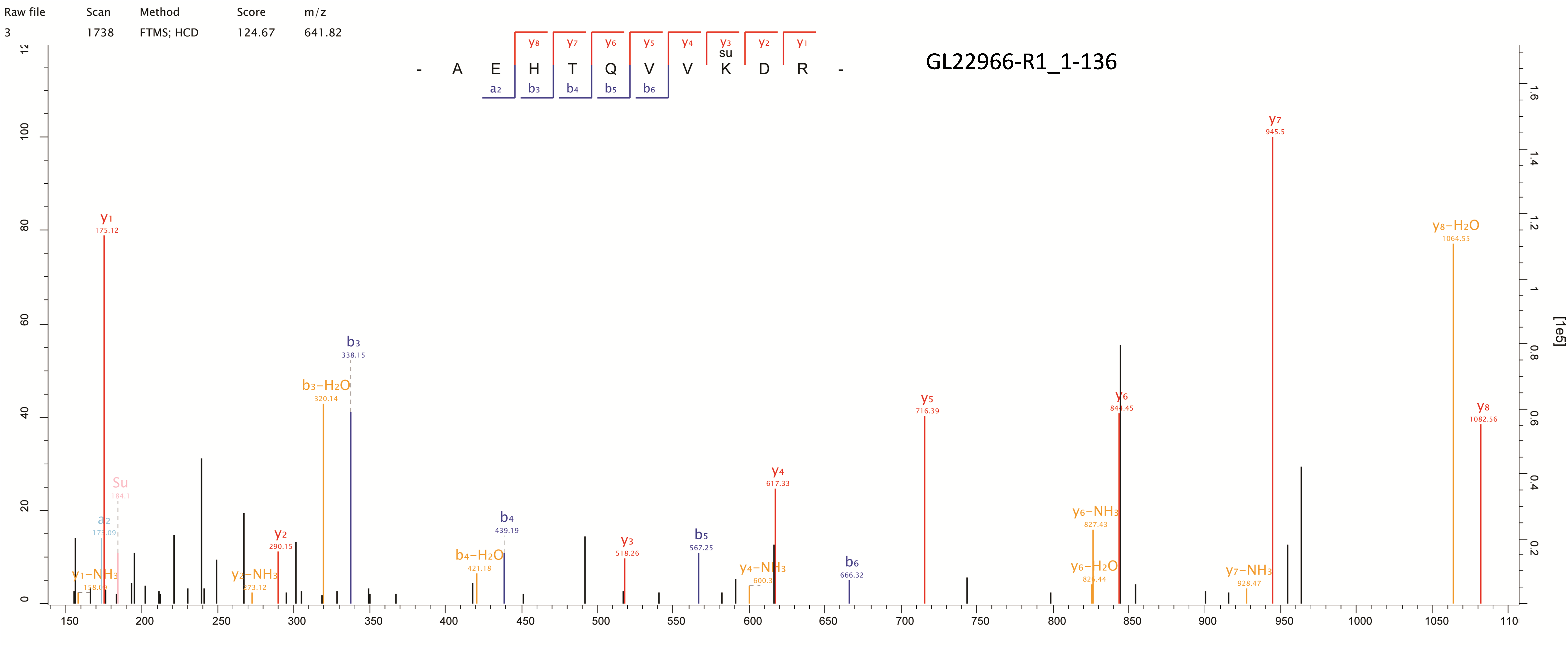
**

**
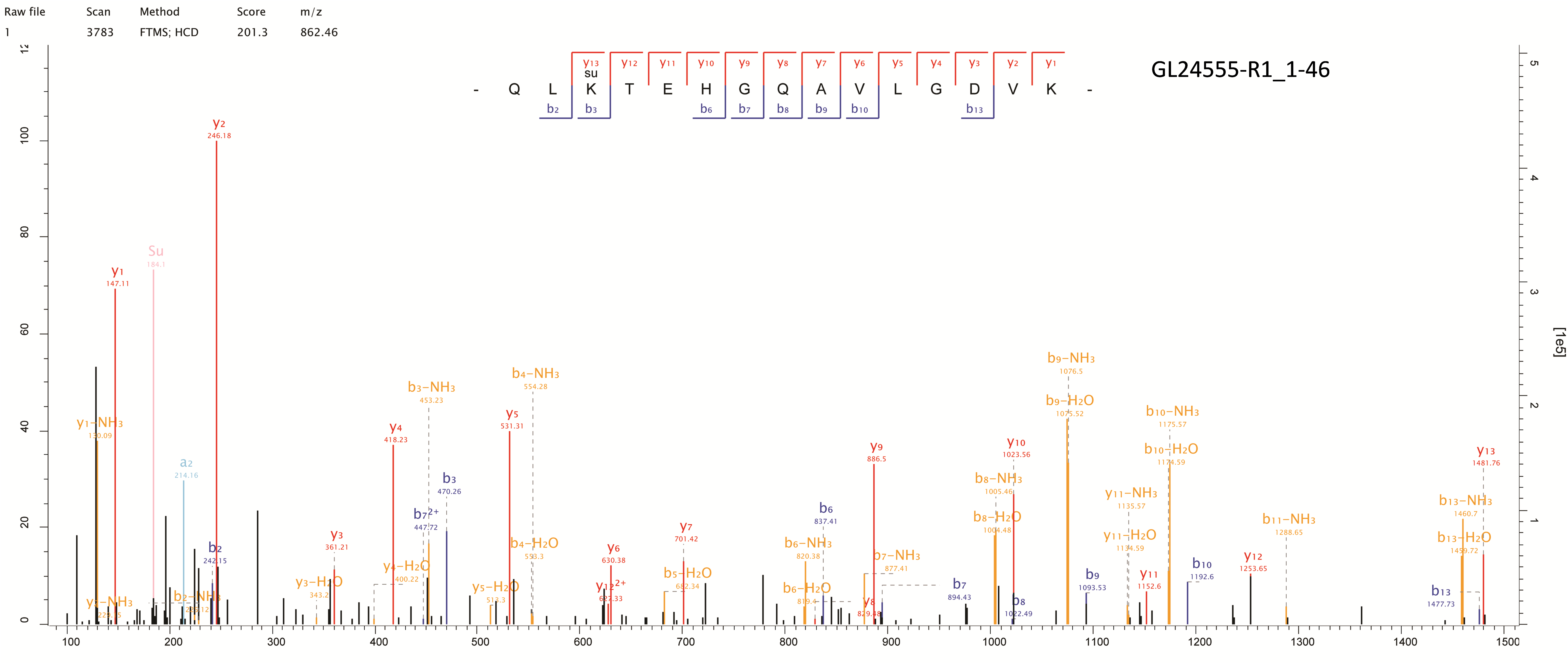
**

**Figure S2.** Western blotting analysis with GAPDH antibody and succinyllysine antibody. Immunoprecipitation of GAPDH was performed with (+) or without (-) GAPDH antibody (Ab) and the eluted proteins were probed with either anti-succinyl lysine antibody (suK) or GAPDH antibody


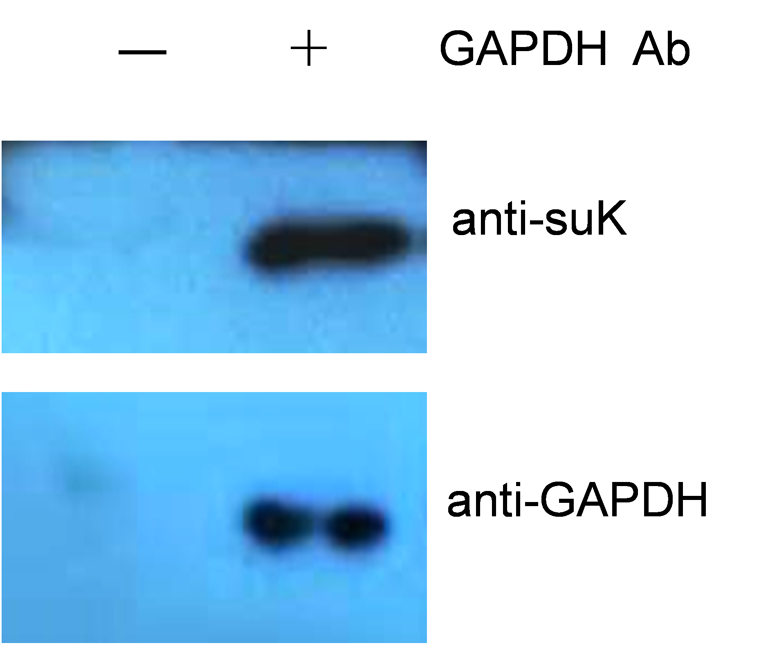


**Figure S3.** GO-based enrichment analysis in terms of cell component (blue bars), molecular function (green bars), and biological process (red bars)

**Figure S4.** Domain enrichment analysis of the succinylproteins.
